# Basal cell carcinoma: 10-year experience with electrochemotherapy

**DOI:** 10.1186/s12967-017-1225-5

**Published:** 2017-05-31

**Authors:** Luca G. Campana, Roberto Marconato, Sara Valpione, Sara Galuppo, Mauro Alaibac, Carlo R. Rossi, Simone Mocellin

**Affiliations:** 1 Department of Surgery, Oncology and Gastroenterology (DISCOG), University of Padova, Padua, Italy; 2 Surgical Oncology Unit, Veneto Institute of Oncology IOV-IRCCS, Via Gattamelata, 64, 35128 Padua, Italy; 3 University of Padova School of Medicine, Padua, Italy; 4 Christie NHS Foundation Trust, Manchester, M20 4BX UK; 5 Radiotherapy Unit, Veneto Institute of Oncology IOV-IRCCS, Padua, Italy; 6 Dermatology Unit, University of Padova, Padua, Italy

**Keywords:** Basal cell carcinoma, Electrochemotherapy, Bleomycin, Skin neoplasms, Neoplasm recurrence

## Abstract

**Background:**

Electrochemotherapy (ECT), by combining manageable cytotoxic agents with short electric pulses, represents an effective palliative skin-directed therapy. The accumulated evidence indicates that ECT stands out as a safe and well-tolerated alternative treatment for patients with multiple or large basal cell carcinoma (BCC), who are not suitable for conventional treatments. However, long-term data and shared indications are lacking.

**Methods:**

In this observational study, we retrospectively analyzed 84 prospectively collected patients with multiple, recurrent or locally advanced BCC who were not candidate for standard therapies and received bleomycin-based ECT according to the European Standard Operative Procedures of ECT, from 2006 to 2016.

**Results:**

Disease extent was local, locally advanced and metastatic in 40 (48%), 41 (49%) and 3 (3%), respectively. Forty-four (52%) individuals had multiple BCCs. Grade 3 skin toxicity after ECT was observed in 6% of cases. Clearance rate was 50% (95% CI 39–61%). Primary presentation (*p* = 0.004), tumor size <3 cm (*p* < 0.001), well-defined borders (*p* = 0.021), absence of tumor ulceration (*p* = 0.001), non-aggressive BCC histology (*p* = 0.046) and age ≤69 years were associated with higher complete response rate. In patients with local BCC, the clearance rate was 72.5 and 85% after one or two ECT cycles, respectively. In the laBCC group, 32 patients (78%) achieved an objective response. Five-year recurrence rate for local and laBCC was 20 and 38%, respectively (*p* ≤ 0.001).

**Conclusions:**

One or two ECT cycles with bleomycin may be a valuable palliative treatment in well-selected patients with multiple BCCs and favorable tumor features. Validation of predictive factors will be imperative to match patients with optimal ECT treatment modalities. Management of laBCC with ECT warrants further investigation.

*Trial registration* ISRCTN14633165 Registered 24 March 2017 (retrospectively registered)

**Electronic supplementary material:**

The online version of this article (doi:10.1186/s12967-017-1225-5) contains supplementary material, which is available to authorized users.

## Background

Basal cell carcinoma (BCC) represents the most common (~80%) form of skin cancer worldwide in white people and its incidence is rising in many countries, although it is not systematically reported in tumor registries [1]. Metastatic spread is extremely rare, but the incidence of locally advanced BCC (laBCC) has been estimated around 8/100,000/year and is associated with substantial morbidity, since most tumors occur in functional areas [2, 3]. Excisional surgery and Mohs staged resection are the most effective treatments for low- and high-risk BCCs, respectively; radiotherapy, curettage and cautery, cryosurgery, carbon dioxide laser, photodynamic therapy, topical immunotherapy represent alternative options in selected cases [4]. Long-term outcomes are crucial in evaluating BCC treatments since it is a slow-growing cancer and recurrence may take long time before being clinically apparent. During the last decade, electrochemotherapy (ECT) has become an appreciated locoregional therapy in the field of dermato-oncology. ECT exerts its anti-tumor effect through the permeabilization of cancer cells to chemotherapy by means of short, high-voltage, electric pulses which destabilize the cell membrane barrier, allowing intracellular access of chemotherapeutic drugs that otherwise would not be able to penetrate the cell effectively [5]. Besides this permissive effect on chemotherapy, ECT exerts a complex vascular disrupting action, which may be usefully exploited when dealing with bleeding tumors [6]. Drugs used in ECT, bleomycin or cisplatin, are cheap, easy to manage and generally safe. According to the European standard operative procedure of ECT (ESOPE), bleomycin can be administered either intratumorally or intravenously, according to the disease burden, while cisplatin can be injected intratumorally, in patients with few and small tumors [7]. Since the publication of the ESOPE in 2006, an increasing number of studies have provided evidence of ECT efficacy on different tumor histotypes [8–12]. On this basis, the National Institute for Health and Care Excellence (NICE) has recently recognized ECT as a safe alternative option for BCC patients (NICE interventional procedure guidance [IPG478], published date: February 2014). However, since the evidence basis is limited and established treatments provides high cure rates, clinicians involved in ECT are encouraged to systematically collect data on case selection, treatment parameters, patient outcomes and, possibly, to submit these data to the International Network for Sharing practices on Electrochemotherapy (InspECT) register (website: http://www.insp-ect.org/) [13]. Although BCC is by far the most frequent skin cancer, clinical experience with ECT is still scarce [10–12, 14–18], probably due to the availability of several established treatment options [19]. In the present study, we sought to examine the feasibility, efficacy and toxicity of ECT in BCC and to gain insights into this potential field of ECT application.

## Methods

### Study population

An observational study was started in 2006 and concluded in 2016 to analyze a 10-year experience with ECT in BCC patients referred to the Veneto Institute of Oncology. All consecutive patients who underwent ECT were included. Eligible subjects were aged 18 years or older and had histologically proven, measurable local, locally advanced or metastatic BCC. Patients with laBCC had to have at least one lesion >2 cm or any size plus ≥2 risk features (21) so that surgical resection was deemed inappropriate (curative resection unlikely, substantial morbidity or deformity anticipated) by a plastic or head and neck surgeon. Moreover, laBCC had to be previously irradiated or, alternatively, radiation therapy had to be contraindicated or inappropriate (e.g. multifocal disease).

In order to be considered for ECT at our center, the patients with local BCC (≤2 cm with a maximum of 1 risk feature) had to present at least two lesions when located in the face or at least three tumors when located in the other anatomical regions. All subject had to be discussed in the frame of a multidisciplinary team. Standard surgical resection was always considered as upfront treatment, when indicated. Other inclusion criteria were ECOG (Eastern Cooperative Oncology Group) performance status 0–2, adequate bone marrow, hemoglobin and renal function (serum creatinine concentration no >1.5 time the upper normal limits), as well as normal respiratory capacity. Exclusion criteria were allergy or previous exposure to bleomycin at maximum recommended cumulative doses, radiological evidence of lung fibrosis, the presence of a pace-maker (for tumors on the chest wall), history of epilepsy, pregnancy, uncontrolled medical illness, concurrent oncological therapies except for endocrine manipulation, and unavailability for follow-up visits. Patient management followed the standards of Good Clinical Practice (GCP). The local Ethic Committee granted approval for the study. All cases were discussed in the frame of a multidisciplinary team meeting. Patients gave written informed consent to the procedure. Anesthesiology evaluation was performed only in patients scheduled under sedation or general anaesthesia. Tumor size (the largest diameter) was clinically measured for later response assessment.

### Treatment

**Fig. 1:**
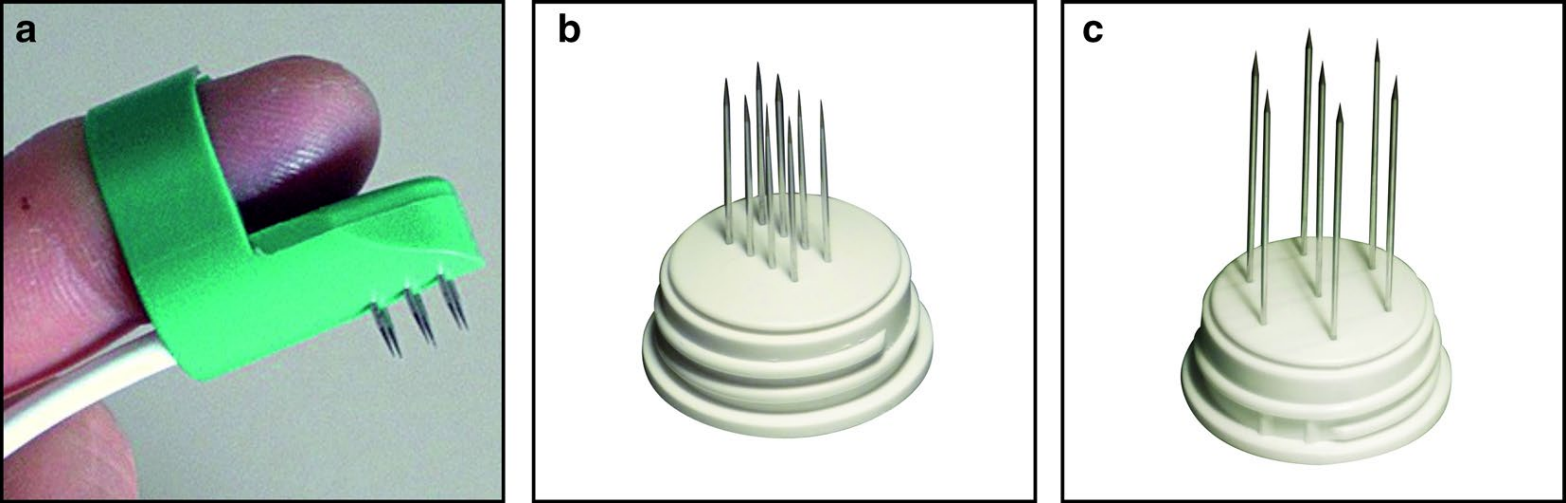
Needle electrodes for tumor electroporation. The “finger” electrode (**a**); the linear array electrode (**b**); and the hexagonal array electrode (**c**)

The ESOPE guidelines were followed in all patients [7]. Accordingly, the type of anaesthesia, the cytotoxic agent and its route of administration were chosen based on patient characteristics, disease extent and tumor anatomical location. Systemic bleomycin (15,000 IU/m^2^) was administered intravenously as a 1-min bolus; alternatively, it was injected intratumorally on each lesion at a dosage of 250–1000 IU/cm^3^ of tumor. Electric pulses were applied by means of a needle electrode (Fig. 1) after 8 or 1 min following intravenous or intratumoral bleomycin administration, respectively. A current of 1.5 and 1.0 Ampere (A) was considered surrogate of adequate tumor electroporation when using a hexagonal array or a linear array needle electrode, respectively [20]. A treatment safety margin around tumor was not consistently adopted in these patients.

### Follow-up and response assessment

Patients were followed up at 1 week and at 1, 2, 6 and 12 months thereafter. Subsequently, they were followed on a 6/12-month basis, according to disease extent, treatment outcome and risk of recurrence. Treated lesions were evaluated at 1 and 2 months by inspection and confirmed local response was classified as complete response (CR), partial response (PR), stable disease (SD) or progressive disease (PD), in accordance with the Response Evaluation Criteria in Solid Tumors (RECIST), adapted for the assessment of superficial disease [8, 9]. The patients who had residual disease (i.e., PR or SD) at 2-month follow-up were offered an additional ECT cycle. Development of additional BCCs also represented an indication to further ECT, when appropriate. Per-patient *treatment success* (assessed after the first and second ECT) required CR achievement and no relevant treatment-induced skin toxicity (i.e., no need of wound dressing). Per-patient *treatment success* at last follow-up required absence of local recurrence/progression, no additional treatments for BCC, and the absence of ECT-induced skin toxicity. Treated tumors were not routinely biopsied for pathological assessment.

### Toxicity evaluation

Local and systemic toxicity was graded according to the Common Terminology Criteria for Adverse Events (CTCAE) [21].

### Statistical analysis

Descriptive data are reported as number and percentage or median and range. Local progression-free survival (LPFS) was the interval from response achievement to disease recurrence or progression. Local response and tumor control were tabulated according to the relevant tumor characteristics and ECT parameters and were analyzed with the χ^2^ test or Mann–Whitney, according to the type of variables. Survival estimates were calculated with the Kaplan–Meier method and patient groups compared with the log-rank test. The analysis was adjusted for clustering of lesions within patients. p < 0.05 was considered statistically significant for two-sided tests. Analysis was done using the R package (v3.0.1, CRAN Project, Vienna).

## Results

### Patient characteristics

Eighty-four patients (53 men, 31 women) with 185 BCCs were treated (Table 1). The median age at entry was 69 years (range 24–89). All except one patient—who was heart-transplanted—had no history of immunosuppression. Patients had a median of two target lesions (range 1–13). Forty-one (49%) patients had a single BCC, while 43 (51%) patients presented with multiple tumors. Median size of the target lesions was 2.0 cm (range 0.5–26.7). BCC distribution according to anatomical site is presented in Additional file 1: Figure S1.

**Table 1:** Patient characteristics (N = 84)

Characteristics	N (%) or median (range)
Sex	
Male/female	53 (63)/31 (37)
Age (years)	69 (24–89)
Comorbidities^a^ (n)	2 (0–6)
Performance status (ECOG)	2 (0–3)
Location of BCC	
Head and neck	52 (62)
Trunk	23 (27)
Limbs	9 (11)
Risk of tumor location	
High^b^	19 (23)
Intermediate^c^	33 (39)
Low^d^	32 (38)
Involvement of multiple anatomical sites	
Yes/no	20 (24)/64 (76)
TNM	
T1	33 (39)
T2^b^	49 (58)
T3	2 (2)
Tumor size	
≤3 cm/>3 cm	52 (62)/32 (38)
Presentation	
Primary/recurrent	42 (50)/42 (50)
Disease extent	
Local	40 (48)
Locally-advanced	41 (49)
Metastatic	3 (3)
Histotype	
Aggressive/non-aggressive^c^	15 (18)/69 (82)
Margins^e^	
Defined/ill-defined	64 (76)/20 (24)
Ulceration	
Yes/no	42 (50)/42 (50)
Bleeding	
Yes/no	11 (13)/73 (87)
Distant metastases	
Yes/no	3 (4)/81 (96)
Need of dressings	
Yes/no	35 (42)/49 (58)
Previous treatments	
Surgical excision	39 (46)
Radiotherapy	20 (24)
Systemic^d^	6 (7)
Other dermatologic treatments^f^	8 (9)
Contraindications to surgery	
None	30 (36)
Multiple recurrences	11 (13)
Expected morbidity	43 (51)
Contraindications to radiotherapy	
None	23 (27)
Previous radiotherapy	19 (23)
Radiotherapy inappropriate	42 (50)

^a^Comorbidities were grouped into the following categories: vascular disease (n = 48, 57.1%); cancer, excluding non-melanoma skin cancer (n = 46, 54.8%), endocrine (n = 25, 29.8%), renal (n = 16, 19.0%), neurologic (n = 9, 10.8%), cardiac (n = 9, 10.8%), hepatic (n = 8, 9.5%), lung (n = 8, 9.5%), gastrointestinal (n = 7, 8.3%); one patient had xeroderma pigmentosum

^b^T2a (n = 32), T2b (n = 17)

^c^Aggressive histotypes included infiltrative and morpheiform BCC; non-aggressive histotypes included superficial and nodular BCC

^d^Vismodegib

^e^The classification was dependent upon the surgeon’s judgment. The BCCs with well-defined borders were those lesions for which the trajectory of a hypothetical surgical incision could unequivocally be individuated. All the other BCCs, in which clinical margins were difficult to distinguish from uninvolved skin were included in the ill-defined margins group (e.g. absence of uniform, well-raised borders, some pigmented BCCs, and some morpheaform BCCs, along with some BCCs previously managed with local treatments)

^f^Cryotherapy (n = 5), PDT (n = 2), imiquimod (n = 1)

### Treatment procedure

One hundred ten ECT cycles were administered (Table 2; Additional file 2: Figure S2). The procedure was applicable in all cases and all treated tumors received adequate electric currents. Eighteen (21.4%) patients received additional skin-directed treatments during the follow-up, due to incomplete response.

**Table 2:** Electrochemotherapy parameters

Characteristics	No (%)
No of ECT cycles	
1	60 (71.4)
2^a^	23 (27.4)
4	1 (1.2)
Anesthesia	
Local	31 (36.9)
Sedation	11 (13.1)
Local + sedation	40 (47.6)
General	2 (2.4)
Route of BLM administration	
Intratumoral	51 (60.7)
Intravenous	33 (39.3)
Electrode^b^	
Finger	12 (14.2)
Linear	46 (54.8)
Hexagonal	26 (31)
Further treatments^c^	
Yes^c^	18 (21.4)
No	66 (78.6)

*ECT* electrochemotherapy, *BLM* bleomycin

^a^The second ECT cycle was administered after a median interval of 3 months (range 2–22)

**Fig. 2:**
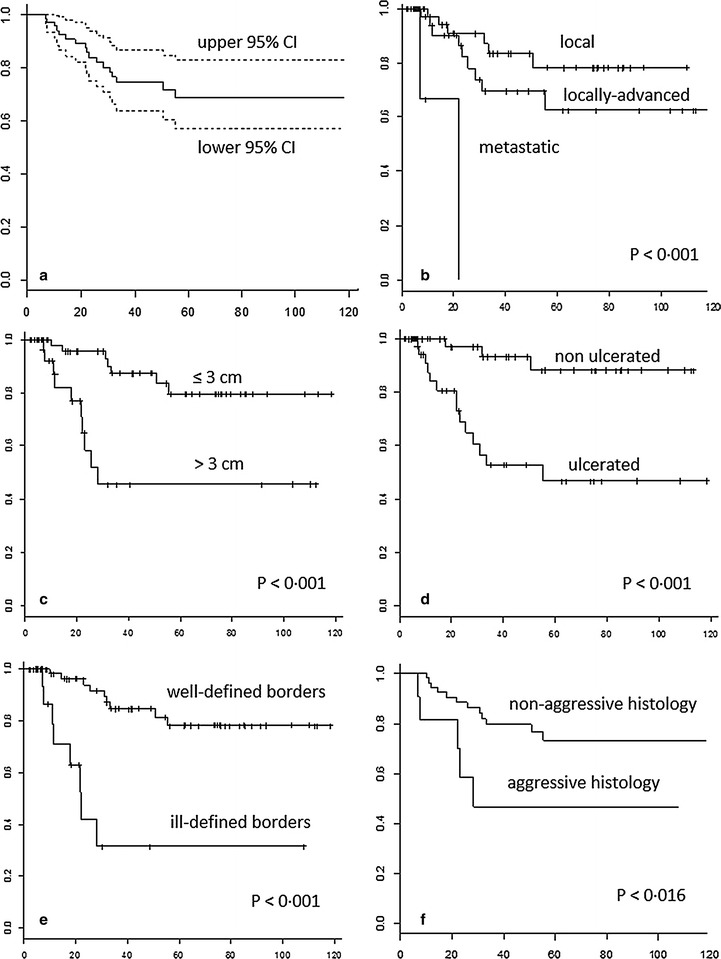
Local tumor control in 84 patients with basal cell carcinoma treated by electrochemotherapy. Local progression-free survival in the whole study population (**a**) and according to disease extent (**b**), tumor size (**c**), ulceration (**d**), tumor borders (**e**), and histology (**f**)

^b^See Fig. 2 for electrode characteristics

^c^Further treatments (administered at least 2 months after ECT) included the following: radiotherapy (n = 9 patients), surgery (n = 4 patients), imiquimod (n = 3 patients), vismodegib (n = 3 patients), local 5-FU (n = 2 patients), cryotherapy (n = 1 patient), laser (n = 1 patient), chemotherapy (n = 1 patient)

### Toxicity

Within the first 24–48 h after treatment, painless erythema and slight edema occurred at the site of electrode insertion in all cases. Within 2 months from ECT, the median grade of patient-reported pain was 1 (range 0–3). We observed some grade of skin toxicity in 53 (63%) patients: G1, n = 35 (42%); G2, n = 10 (12%); G3, n = 5 (6%), G4, n = 3 (4%); all G3 and G4 toxicities were observed in patients who had ulcerated laBCC at baseline. Sixteen (19%) patients experienced some form of ECT-induced skin ulceration (G1, n = 14 patients; G2, n = 2 patients). Ulceration appeared within the first week. Complete wound healing by second intention required an average of 4–8 weeks. One patient experienced a wound infection on a locally advanced BCC of the trunk, which responded to oral antibiotics. At the last follow-up, none, except one, of these patients needed wound dressing. Only 6 out of 42 patients with ulcerated BCC at baseline, achieved wound healing at 2 months, however at the last follow-up 15/84 (18%) patients were requiring wound dressing for BCC, compared with 35/84 (42%) at baseline (*p* < 0.001). In patients with primary BCC (n = 42), the distribution of skin toxicity observed within 2 months after the first ECT was as follows: G0, 23 (54.8%); G1, 15 (35.7%); G2, 3 (7.1%); G3, 1 (2.4%). In patients with recurrent BCC (n = 42), skin toxicity was as follows: G0, 8 (19%); G1, 20 (47.6%); G2, 7 (16.7%); G3, 4 (9.5%); G4, 3 (7.1%).

### Tumor response

Overall response rate after the first ECT cycle was 85.7% (72/84 patients) (95% confidence interval [CI] 78–93%). CR rate was 50% (42/84 patients) (95% CI 39–61%) (Additional file 3: Figure S3, Additional file 4: Figure S4). Treatment outcomes according to the investigated variables are reported in Table 3. Younger (≤69 years) patients achieved a higher CR rate compared with older ones. The CR rate was significantly higher in patients with primary than recurrent BCC as well as in patients with local BCC compared with laBCC. In BCCs ≤3 cm the CR rate was 69.2%, while in tumors >3 cm it was 18.7% (*p* < 0.001) (Additional file 5: Figure S5). Well-defined borders, non-aggressive histology and absence of ulceration also were significantly associated with CR achievement. In the subgroup of 24 patients who underwent a second ECT cycle, tumor response was as follows: CR, 11 patients (45.8%); PR, 11 patients (45.8%); SD, 2 patients (8.4%). Overall, after the second ECT cycle CR rate increased from 50% (42/84) to 63% (53/84). In patients with local BCC (n = 40) tumor response after the first ECT cycle was as follows: CR, 29; PR, 10; SD, 1. Six of these patients underwent a second ECT cycle, achieving CR (n = 5) and PR (n = 1). Accordingly, in patients with local BCC, the clearance rate after the first and the second ECT cycle was 72.5 and 85%, respectively. Finally, we observed a higher CR rate in patients who received intratumoral compared with intravenous bleomycin (62.7% vs 32.3%, *p* = 0.005). Moreover, CR rate decreased with the increasing electrode size: finger 75%, linear array 54.3%, hexagonal array 30.8% (*p* = 0.027) (Table 3).

**Table 3:** Treatment outcome according to tumor characteristics in 84 patients with basal cell carcinoma treated by electrochemotherapy

Variable	Response rate, *n* (%)	*p*	CR rate *n* (%)	*p*	LPFS (mos), median (range)	*p*	5-year recurrence rate, %
Age at first ECT		0.756		*0.048*		0.077	
≤69 (n = 42)	37 (88.1%)		27 (64.3%)		35.0 (2.2–120.1)		–
≥70 (n = 42)	35 (83.3%)		15 (35.7%)		23.3 (3.6–114.8)		
PS (ECOG)		0.760		0.512		0.134	
0–1 (n = 40)	35 (87.5%)		18 (45%)		39.7 (2.2–120.1)		–
2–3 (n = 44)	37 (84.1%)		24 (54.5%)		24.7 (6.8–114.5)		
No of comorbidities		1		0.826		0.183	
0–2 (n = 46)	39 (84.8%)		24 (52.2%)		39.0 (2.2–114.8)		–
3–6 (n = 38)	33 (86.8%)		18 (47.4%)		27.2 (2.2–120.1)		
Presentation		0.244		*0*.*004*		0.141	
Primary (n = 42)	38 (90.5)		28 (66.7)		34.2 (2.2–118.4)		–
Recurrent (n = 42)	34 (80.9)		14 (33.3)		29.1 (3.5–113.1)		
BCC extent		*0.001*		<*0.001*		<*0.001*	
Local (n = 40)	39 (97.5)		29 (72.5)		38.9 (2.2–110.0)		20
Locally-adv (n = 41)	32 (78.0)		13 (31.7)		23.0 (3.5–118.4)		38
Aggressive histology		*0.006*		*0.046*		*0.016*	
Yes (n = 15)	9 (60)		4 (26.7)		21 (3.5–107.9)		52
No (n = 69)	63 (91.3)		38 (55.1)		33·7 (4.7–118.4)		24
T size		*<0.001*		<*0.001*		<*0.001*	
≤3 cm (n = 52)	52 (100)		36 (69.2)		44.3 (2.2–118.4)		20
>3 cm (n = 32)	20 (62.5)		6 (18.7)		11.5 (3.5–112.5)		58
Ill-defined borders		<*0.001*		*0.021*		<*0.001*	
Yes (n = 20)	12 (60)		5 (25)		11.2 (3.5–107.9)		68
No (n = 64)	60 (93·7)		37 (57.8)		40.75 (2.2–118.4)		22
Location risk^a^		0.728		0.436		0.212	
Low (n = 32)	29 (90.6)		19 (59.4)		31 (15.5–118.4)		–
Intermediate (n = 33)	26 (78.8)		14 (42.4)		21.0 (4.7–107.9)		
High (n = 19)	17 (89.5)		9 (47.4)		48.7 (6.7–75.3)		
Ulceration		*0.005*		*0.001*		<*0.001*	
Yes (n = 42)	31 (73.8)		13 (30.9)		21.3 (3.5–118.4)		52
No (n = 42)	41 (97.6)		29 (69)		43.0 (2.2–113.1)		12
TNM		<*0.001*		<*0.001*		0.174	
T1 (n = 32)	32 (100)		26 (81.2)		41.5 (2.2–113.1)		–
T2 (n = 50)	40 (80)		16 (32)		22.9 (3.5–118.4)		
T3 (n = 2)	0 (0)		0 (0)		7.0 (6.8–7.3)		
BLM route		*0.002*		*0.005*		*0.007*	
i.t. (n = 51)	49 (96.1)		32 (62.7)		41.7 (2.2–118.4)		–
i.v. (n = 33)	23 (69.7)		10 (30.3)		14.5 (3.5–112.5)		
Electrode		*0.05*		*0.027*		0.423	
Finger (n = 12)	12 (100)		9 (75)		41.5 (17.3–87.9)		–
Linear (n = 46)	41 (89.1)		25 (54.3)		26.8 (2.2–118.4)		
Hexagonal (n = 26)	19 (73.1)		8 (30.8)		20.6 (3.5–113.1)		

Italic values indicate significance of *p* value (*p* < 0.05)

*CR* complete response, *LPFS* local progression-free survival, *mos* months, *PS* performance status, *ECOG* Eastern Cooperative Oncology Group, *BCC* basal cell carcinoma, *adv* advanced, *BLM* bleomycin

^a^Low risk locations: trunk and extremities; intermediate risk locations: cheeks, forehead, scalp, neck; high risk locations: central face, eyelids, peri-orbital, nose, lips, chin, mandible, ear, pre-/post-auricular, genitalia, hands and feet

Among the BCCs of the head and neck region, the clearance rate according to anatomical location was as follows: ear (n = 16) 25%; periorbital (n = 2) 50%; nose (n = 19) 52.6%; neck (n = 7) 57.1%; cheek (n = 22) 59.1%; scalp (n = 40 tumors) 62.5%; forehead (n = 9) 66.7%; lip (n = 3) 100%. We observed a significantly higher CR rate among BCCs located on the scalp when compared to all the other locations of the head and neck district (*p* = 0.03).

### Tumor control

Median follow-up time was 49.2 months (range 3.6–121.1). Treatment failure was documented in 17 out of the 84 (20.2%) patients, after a median of 22 months (range 6.8–55.2). Eleven of these 17 patients relapsed after a single ECT session (3 patients achieved CR, 8 PR); the remaining 6 patients had initially received 2 ECT cycles (4 patients achieving PR, 2 SD). The remaining 67 patients were free from local recurrence or progression at last follow-up. Of these 67 patients, 6 were still requiring some kind of wound dressing at the last evaluation (Additional file 5: Figure S5). Five of these patients received a single ECT (4 PRs, 1 SD) and one patient 2 ECT cycles, achieving SD and then PR; the latter patient received also imiquimod and radiotherapy after ECT, achieving PR.

Treatment success rate following the first and the second ECT cycle as well as at the last follow-up was 40.5% (34/84 patients), 51.2% (43/84 patients) and 65.5% (55/84 patients), respectively (p = 0.005). Five-year LPFS was 70% (95% CI 58–82%). The following clinical and procedural factors were associated with better tumor control: local disease extent (*p* < 0.001), non-aggressive histology (*p* = 0.016), tumor size ≤3 cm (*p* < 0.001), T1 stage (*p* = 0.036), well-defined borders (*p* < 0.001), absence of ulceration (*p* < 0.001) (Table 3; Fig. 2). Finally, we observed that patients who received intratumoral bleomycin achieved better tumor control compared with those treated with intravenous bleomycin (median LPFS, 41.7 months [range 2.2–118.4] vs 14.5 months [range 3.5–112.5], *p* = 0.007). Moreover, median LPFS decreased with the increasing size of the electrode: finger 41.5 months (range 17.3–87.9), linear array 26.8 (range 2.2–118.4), hexagonal array 20.6 (range 3.5–113.1) (*p* = 0.423).

Twenty-four patients (28.6%) received a second ECT cycle. In this group, the recurrence rate was 25% (6 out of 24 patients), compared with 18.3% (11 out of 60 patients) in the group that received a single ECT, but this finding likely reflects the more unfavorable characteristics (larger size) of tumors that were treated twice. When splitting patients according to disease extent (e.g. local vs laBCC), the benefit of retreatment was more evident in patients with local BCC. In this subgroup (n = 40 patients), the recurrence rate was 17.6% (6/34 patients) after a single ECT cycle and 0% (0/6 patients) after two ECT cycles (*p* = 0.264). In patients with locally-advanced disease, the recurrence rate was 19.2% (5/26 patients) and 26.7% (4/15 patients) after one or two ECT cycles, respectively (*p* = 0.579). The recurrence rate according to BCC presentation was as follows: primary BCC, 14.3% (6 out 42 patients); recurrent BCC, 26.2% (11 out 42 patients), *p* = 0.174. Accordingly, 5-year recurrence rate for local and laBCC were 20 and 38%, respectively.

### Patient outcome

During follow-up, 16 (19%) patients were diagnosed with additional skin cancers. Ten out 84 (12%) patients developed further BCCs and four of them received additional ECT cycles (3 achieved CR, 1 PR). Additional skin cancer diagnoses included melanoma (n = 7) and SCC (n = 3).

## Discussion

As the prevalence of BCC rises with aging of the population, clinicians involved in BCC treatment face an increasing number of challenging scenarios. These are the patients with laBCC, hereditary basal cell naevus syndrome, and those with multiple or recurrent BCCs, who are compelled to undergo multiple interventions. Although these patients represent only a small proportion of the overall BCC population, nonetheless the burden of disease prompts for new evidence-based therapies especially for those individuals who are not amenable to surgical treatment (i.e., excision would be disfiguring or unpractical). In the present study, which is based on the largest available BCC series managed by ECT, we evaluated 84 subjects who were treated according to a standardized protocol (ESOPE) [7]. Since ECT indications were restrictive, our study population has at least two peculiar characteristics: the high number of subjects with relevant comorbidities (Table 1), and the high percentage of laBCCs (49%). Since the presence of comorbidities has been shown to impact on the survival of elderly patients, especially for less lethal cancers [22], a minimally invasive, low-demanding treatment modality can be considered a reasonable alternative in well-selected cases. The simplicity of treatment application, the ability to simultaneously treat multiple lesions (Additional file 4: Figure S4) coupled with high patient tolerability, represent potential advantages of ECT in patients with multifocal or laBCC or in presence of diseases that limit the number of practicable therapies [17].

Electrochemotherapy was successfully applied in all cases, with no serious adverse events. Overall, we observed a 50% CR rate after a single ECT cycle, with significantly higher clearance rates in younger patients with primary BCC presentation, local tumor extent, small tumor size, well-defined borders, absence of ulceration and non-aggressive histology. Overall, local recurrence occurred in 20% of patients (primary BCC, 14%; recurrent BCC, 26%) and ECT-induced toxicity was observed in 19% of patients, being mild and transient in most cases. Noteworthy, we reported a significant decrease (from 42 to 18%) in the fraction of patients who required wound dressing during the follow-up. This is a relevant observation since BCC, and especially advanced BCC, is associated with a significant disease burden and health care resource utilization. The same parameters that were associated with CR achievement—with the exception of BCC presentation and TNM classification—were also associated with longer tumor control. Some of these variables support the notion of tumor response dependency upon tumor size in ECT, as previously observed by other authors [8, 9, 23]. Interestingly, BCCs with ill-defined borders were less responsive to treatment. For this reason, we advocate the adoption and quantification of predefined treatment safety margins as a valuable measure to minimize the risk of recurrence from subclinical tumor deposits [13]. In this regard, general anesthesia—together with no previous surgical treatment—was found to be associated with significantly higher CR rate in patients with non-melanoma head and neck skin cancers who underwent ECT according to a recent multicenter phase II study including 55 individuals, of whom 24 with BCC [24]. This observation raises the hypothesis that general anesthesia may ensure better treatment tolerability by patients and thus a more accurate tumor coverage with electrode placements during the procedure.

In addition, in our study the patients with aggressive BCC histotypes proved to be less responsive; therefore, BCC-specific histopathological parameters should be taken into account in future studies in order to refine patient selection. Finally, we correlated treatment outcome with ECT procedural parameters, but these observations are likely biased by differences in BCC size [7]. Accordingly, the identification of the best route of drug administration as well as the most suitable electrode type in ECT will require a dedicated study with more homogeneous tumor groups.

It should be noted that currently available ECT guidelines [7] mainly refer to the feasibility of the procedure itself, but are not informative about the appropriateness of treatment indication or the optimal number of treatment cycles. These aspects need to be elucidated in future studies. According to our experience, a second ECT cycle increased the CR rate from 50 to 63% and retreatment was more advantageous in patients with local BCC, in whom CR rate increased from 72.5 to 85%. However, numbers are small and these findings should be evaluated with caution. For instance, the benefit of retreatment has been observed in BCC treated by 5-aminolaevulinic acid photodynamic therapy. In a longitudinal, non-randomized study on 44 patients with primary or recurrent BCC, Christensen et al. [25] reported a CR rate of 60 and 87% after one or two treatment sessions, respectively. For the time being, retreatment with ECT seems a reasonable option in patients with small BCCs in order to consolidate response duration, particularly when risk factors such as aggressive histology, ill-defined borders or ulceration are present (Table 3).

Our results appear less satisfactory as compared to the literature data on BCC treatment with ECT [24]. It is remarkable that no comparative trials have been carried out so far and no cost-analyses are available. The bulk of the literature consists of small observational series, often including heterogeneous skin cancers [10, 12, 14–18, 24]. In an early trial, Glass et al. [16] reported a 98% response rate after one or more ECT cycles with intralesional bleomycin and no recurrences detected after 18 months. However, this trial, which adopted a different electroporation protocol, enrolled exclusively patients with sporadic BCC and low tumor size (mean diameter 0.91 cm, range 0.37–2.1). A recent meta-analysis assessing 47 prospective studies on patients with cutaneous metastases—mainly from melanoma and breast cancer—treated by skin-directed therapies (including ECT, radiation, photodynamic, intralesional and topical therapies) showed sustained response rates across treatments with G ≥ 3 dermatological toxicity in less than 6% of patients [26]. More recently, a European and an Italian multicenter studies (both adopting the ESOPE protocol), have reported CR rates of 91 and 66.7%, respectively, in BCC patients, although with a short follow-up (6 and 13.9 months, respectively) [10, 11]. In 2016, Rotunno et al. reported a 75% CR rate in patients with head and neck BCC treated by ECT. The majority (42%) of these tumors (whose median size was 24 mm) were located on the scalp and half of patients required at least 2 ECT cycles [24]. An ongoing randomized trial is currently evaluating treatment durability of surgical excision versus ECT in patients with primary BCC. An interim analysis, based on 86 patients at 3-year follow-up, indicates comparable efficacy, as indicated by local disease-free progression of 97 and 92%, respectively (*p* = 0.37) [27].

Recurrence rates in primary and recurrent BCC treated by conventional treatments can be summarized as follows: excisional surgery, 2–17.4% [28–31]; Mohs surgery, 1.0–5.6% [28, 32]; curettage and electrodessication, 7.7–40% [28, 33]; cryotherapy, 7.5–13% [28, 32]; radiotherapy, 7.4–16% [28, 32, 34, 35]. Topical immunotherapy with imiquimod ensures 5-year recurrence rate around 20% in small superficial BCC [36]. Finally, recurrence rate after photodynamic therapy, in its various forms of ALA-PDT (aminolevulinic acid photodynamic therapy) and MAL-PDT (methyl aminolevulinate photodynamic therapy), ranges to 18–22% [37, 38]. Taken together, these data indicate that ECT has an acceptable toxicity profile, but a higher recurrence rate, especially when compared with excisional and Mohs surgery. In the present study, 5-year recurrence rate in local and laBCC was 20 and 38%, respectively. There are same possible explanations for these findings. First, we cannot exclude suboptimal treatment delivery. ECT exerts its effect when tumors are simultaneously exposed to chemotherapy and electric fields. In this regard, BCC poses peculiar challenges. More than 60% of our patients had tumors located on the head and neck region, where direct drug injection can lead to inhomogeneous distribution or to spillage from the injection site, as previously observed [39]. Further, we hypothesize that the diffusion of chemotherapy in tumor tissue may have been hampered by the presence of scars from previous treatments, thus leading to inhomogeneous drug distribution [24]. Second, it is possible that some tumors could have been covered sub optimally by electric pulses. Since 36.9% of patients were managed under local anesthesia only, it is likely that sedation or general anesthesia would have allowed a deeper (e.g. in laBCCs) or wider (e.g. in BCCs with ill-defined borders) electrode application [24]. In support of this hypothesis, it is worthy to note that in the present series 20 out of 84 patients had tumors with clinically ill-defined borders and that these tumors had the highest recurrence rate (Table 3). A third explanation may reside in tumor biology. In fact, the fraction of replicating cells in BCC is low [40] and cytotoxic agents, which selectively kill the actively replicating cells, exert their action on a small fraction of them. Finally, the relatively long follow-up of our study allowed for the detection of late recurrences.

Surgical treatment definitely represents the preferred options for BCC, with conventional excision being sufficient in most primary BCCs and Mohs micrographic surgery being the most effective in high risk or recurrent BCCs of the face, where it is associated with 5- and 10-year recurrence rates of 2.1–5.2% and 3.9–4.4%, respectively [41–43]. Among non-surgical options, radiotherapy represents a consolidated and effective alternative [3, 4, 34, 35], although logistical barrier may be a limitation. In selected cases, ECT may be a rapid, easy to apply treatment, although the broad spectrum of BCC presentation poses peculiar challenges to its application. In order to maximize the efficacy to toxicity ratio, clinicians should aim to select the most appropriate ECT treatment modality (type of anaesthesia, route of drug administration and electrode geometry). In theory, systemic chemotherapy, coupled with use of a large needle electrode (i.e., the hexagonal array, Fig. 1a) allows homogeneous tumor tissue exposure to chemotherapy and complete tumor electroporation. However, this should be weighed against possible side effects. Systemic bleomycin can be associated, although in very rare cases, with lung toxicity (especially in patients >70 years of age) [44], and the application of a large needle electrode can increase skin injury due to its greater invasiveness [39]. Furthermore, clinician and patients could be reluctant to systemic chemotherapy for the treatment of a localized, slow growing tumor. ECT proved to be effective in BCCs located on the midface, where treatment could be potentially challenging due to the presence of aesthetic and functional structures. According to a well-documented series of three patients with BCC of 0.5–1.0 cm^2^ affecting the peri-ocular region, treatment with ECT is feasible, safe and associated with acceptable scarring [18].

Interestingly, we observed a 31.7% CR rate in laBCC (Table 3), with appreciable tumor shrinkage also in partial responders (Additional file 5: Figure S5). Only a few studies of chemotherapy in BCC have been published and most of them found a relative unresponsiveness, except for limited success with cisplatin [45]. We did not perform cisplatin-based ECT, since, according to the ESOPE, this drug is currently codified for intratumoural injection only, and specifically in small size tumors [7]. Although the laBCC field has successfully entered the era of targeted therapy, locoregional treatments continue to be worth of consideration, particularly in the frame of integrated strategies. CR rates ranging from 21 to 34% have been consistently observed with the hedgehog inhibitor vismodegib, with median PFS ranging from 9.5 to 24.5 months [46, 47]. However, tolerability still represents a tangible obstacle in continuation of therapy, with 10–25% of patients requesting to stop treatment and 36% discontinuing due to adverse events (mainly muscle spasms, alopecia and dysgeusia) [46, 47]. In this context, local treatment with ECT could be rationally associated or combined (e.g. during the so-called *drug holidays*) with target therapy in order to maximize or consolidate tumor response. However, for the time being, the limited experience does not allow general recommendations, therefore prospective trials are warranted to assess the impact of ECT in advanced BCC.

Although non-comparative, the present study has some strengths. It is based on a relatively large series. It included BCC-specific parameters (histological subtypes, tumor borders, ulceration) in order to personalize treatment indication. It was conducted at a center, which is long acquainted with the procedure [8, 9, 11, 39]. Finally, it has a relatively long follow-up, which is crucial for assessing late recurrences [28, 43]. Nevertheless, we also acknowledge a number of limitations. First, this study is not comparative. Second, we included patients with heterogeneous BCC types and adopted a conservative definition for laBCC. Third, tumor response was clinically assessed, and thus possibly overestimated, with no independent review assessment. Fourth, we did not apply treatment safety margins around the target tumor in all cases. Finally, patient-reported outcomes and functional as well as cosmetic results were not included. It is desirable that disease-specific questionnaires will be incorporated in future studies together with 5- and 10-year follow-up data.

## Conclusions

We tested ECT as a palliative alternative treatment in a challenging cohort of patients with relevant comorbidities and a high percentage of recurrent or laBCC. One or two ECT cycles eradicated treated tumors in 50 and 63% of patients, respectively, with limited toxicity. Large tumor size, recurrent presentation, locally advanced disease, aggressive BCC histology, ill-defined borders and tumor ulceration were limiting factors to CR achievement. These factors can be overcome by a second ECT cycle, at least in patients with small BCC. In those with laBCCs, ECT provides effective tumor control, but the optimal number of treatment cycles, patient compliance and possible combined approaches with targeted therapy remain to be established. Validation of predictive factors is imperative in order to match each single BCC patient with the most suitable ECT treatment modalities. Ongoing and future trials will help to clarify the role of ECT in the treatment algorithm of BCC patients.

## Additional files

**Additional file 1: Figure S1.** Distribution of basal cell carcinomas according to anatomical location.

**Additional file 2: Figure S2.** Outcome of 84 patients with basal cell carcinoma treated with electrochemotherapy.

**Additional file 3: Figure S3.** Treatment with electrochemotherapy of a recurrent (after previous cryotherapies and surgical resection) nodular basal cell carcinoma of the scalp in a patient with multiple BCCs and history of active inflammatory bowel disease. Baseline (**a**); partial response after first electrochemotherapy cycle (**b**); patient outcome with persisting complete regression after 2.5 years (**c**).

**Additional file 4: Figure S4.** A primary basal cell carcinoma of the trunk in a patient with concomitant in-transit metastases from melanoma on the lower limb. Both basal cell carcinoma and lower limb in-transit metastases were simultaneously treated with electrochemotherapy; bleomycin was administered intravenously – according to the European Standard Operative Procedure of Electrochemotherapy (ESOPE)-, due to the concomitant presence of multiple in-transit metastases from melanoma. Baseline presentation (**a, b**); one month after electrochemotherapy (**c**); two-year and six-month follow-up showing long-lasting complete response (**d**).

**Additional file 5: Figure S5.** Recurrent, locally advanced basal cell carcinoma of the back in a 55 years-old woman with a synchronous lung metastasis. The latter was biopsied under CT-scan guidance. The pathology report indicated a metastasis from basosquamous carcinoma. The patient received anti-hedgehog therapy (vismodegib) with partial response; side effects associated with systemic treatment were tolerated for eight months and the patient finally refused further administrations. Therefore, stereotactic radiosurgery was performed on the lung metastasis. As to the basal cell carcinoma on the trunk, several surgical resections were previously attempted, also followed by external radiotherapy. The tumor was managed with four bleomycin-based ECT cycles, by using both intravenous and intratumoral chemotherapy. (a) Baseline presentation; (b) two-year follow-up indicating partial response with appreciable wound healing; tumor response was pathologically assessed by means of six punch biopsies, of which four confirmed tumor clearance (lower insert) and two showed residual disease (upper insert).

## Abbreviations

ECT: electrochemotherapy
BCC: basal cell carcinoma
laBCC: locally advanced basal cell carcinoma
ESOPE: European Standard Operative Procedures of Electrochemotherapy
NICE: National Institute for Health and Care Excellence
InspECT: International Network For Sharing Practices of Electrochemotherapy
ECOG: Eastern Cooperative Oncology Group
GCP: Good Clinical Practice
RECIST: Response Evaluation Criteria in Solid Tumors
CR: complete response
PR: partial response
SD: stable disease
PD: progressive disease
CTCAE: Common Toxicity Criteria for Adverse Events
ALA-PDT: aminolevulinic acid photodynamic therapy
MAL-PDT: methyl aminolevulinate photodynamic therapy
