# Supplementary figures and images for: Basal cell carcinoma: 10-year experience with electrochemotherapy
Source: J Transl Med. 2017 May 31;15:122. doi: 10.1186/s12967-017-1225-5 (PMC5452531; doi:10.1186/s12967-017-1225-5)

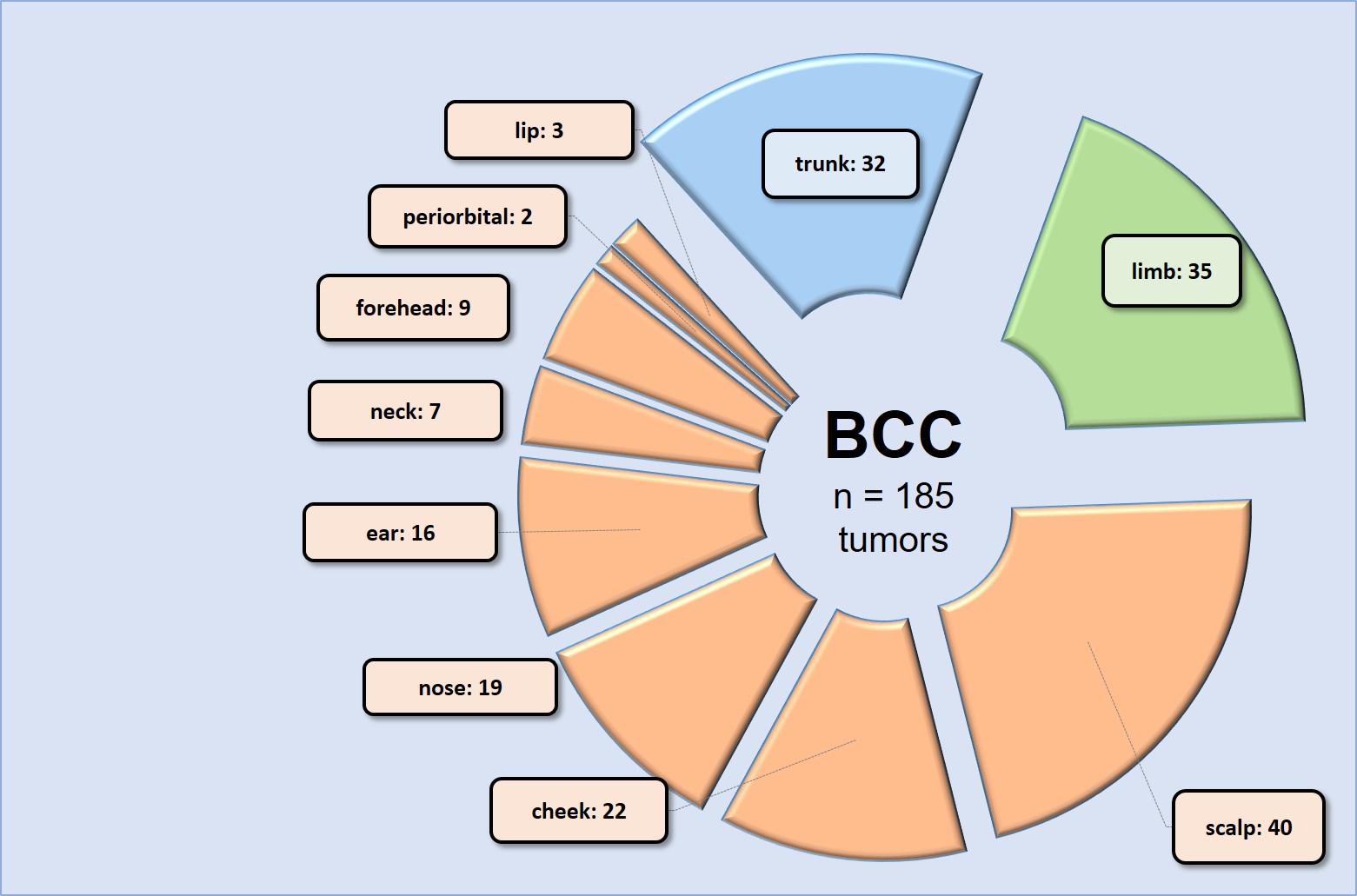

Supplement: Supplementary file 1 — Additional file 1: Figure S1. Distribution of basal cell carcinomas according to anatomical location. [file 12967_2017_1225_MOESM1_ESM.jpg]

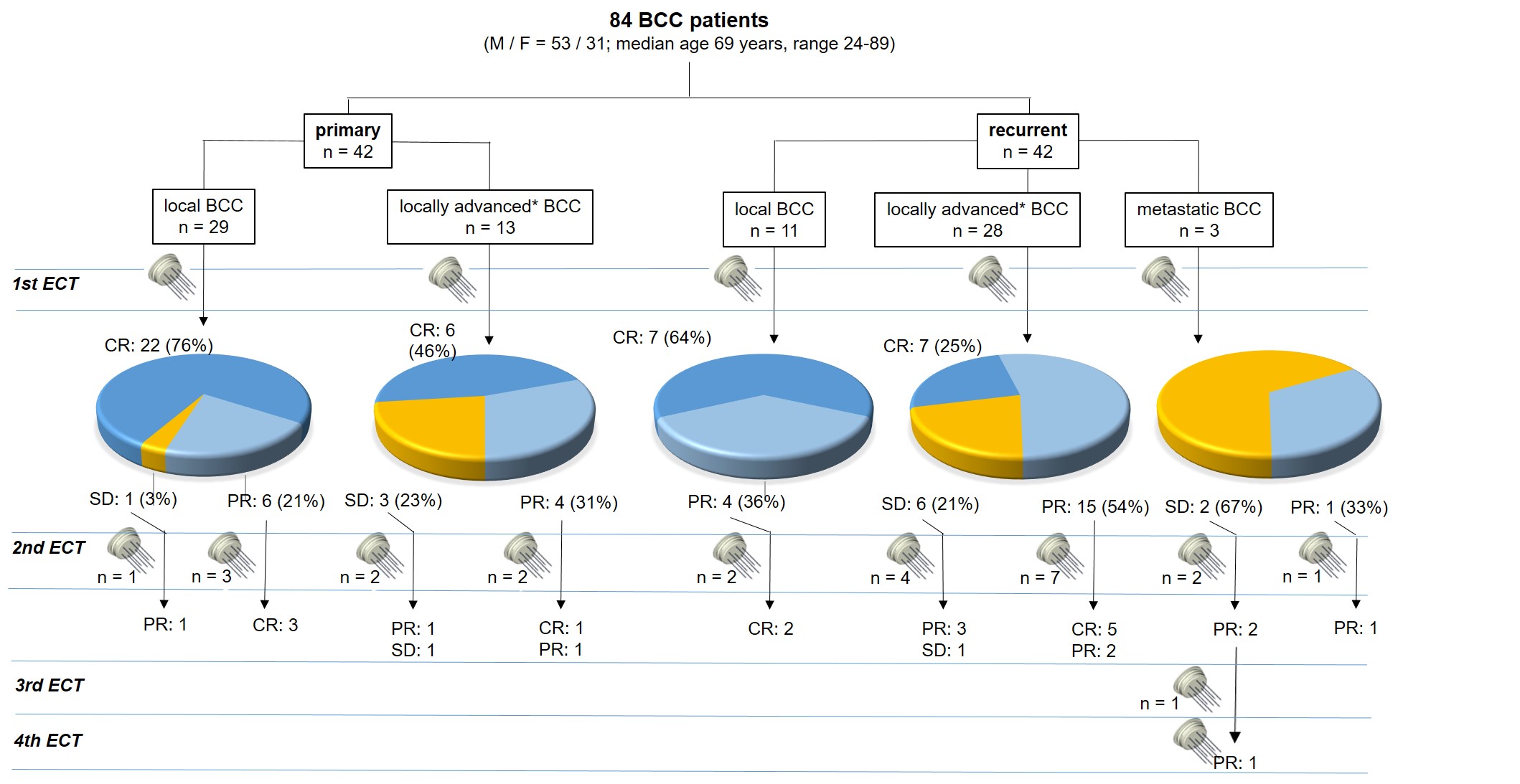

Supplement: Supplementary file 2 — Additional file 2: Figure S2. Outcome of 84 patients with basal cell carcinoma treated with electrochemotherapy. [file 12967_2017_1225_MOESM2_ESM.jpg]

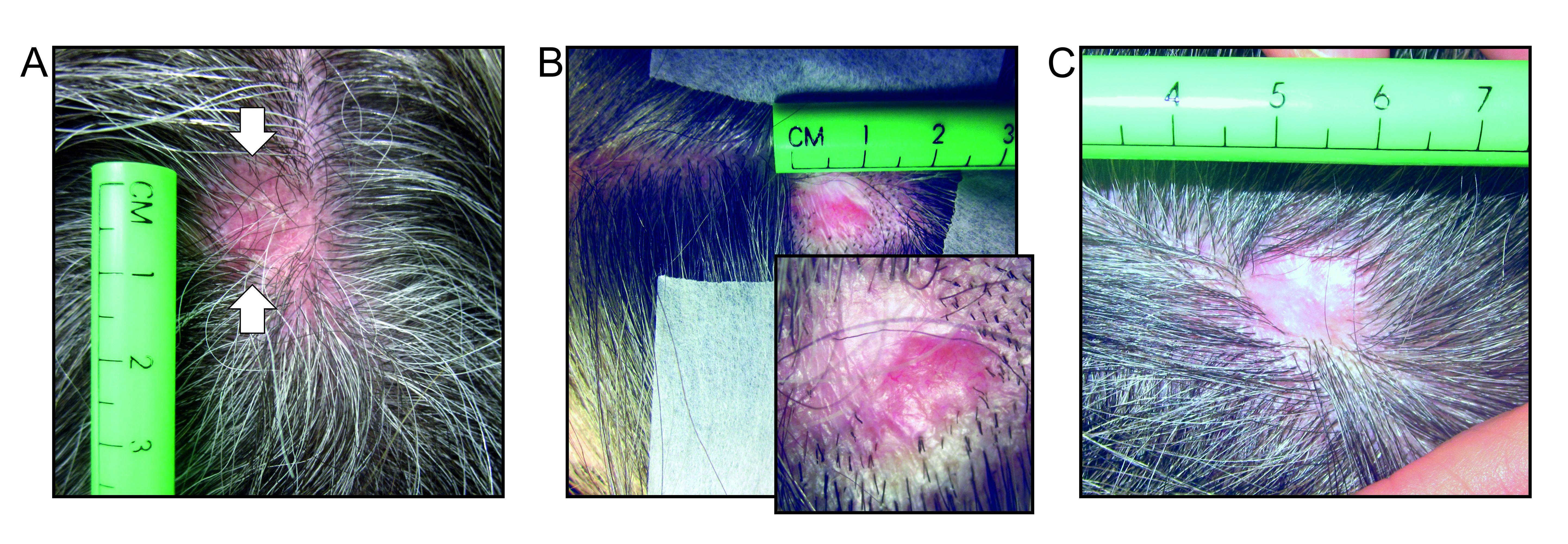

Supplement: Supplementary file 3 — Additional file 3: Figure S3. Treatment with electrochemotherapy of a recurrent (after previous cryotherapies and surgical resection) nodular basal cell carcinoma of the scalp in a patient with multiple BCCs and history of active inflammatory bowel disease. Baseline (a); partial response after first electrochemotherapy cycle (b); patient outcome with persisting complete regression after 2.5 years (c). [file 12967_2017_1225_MOESM3_ESM.jpg]

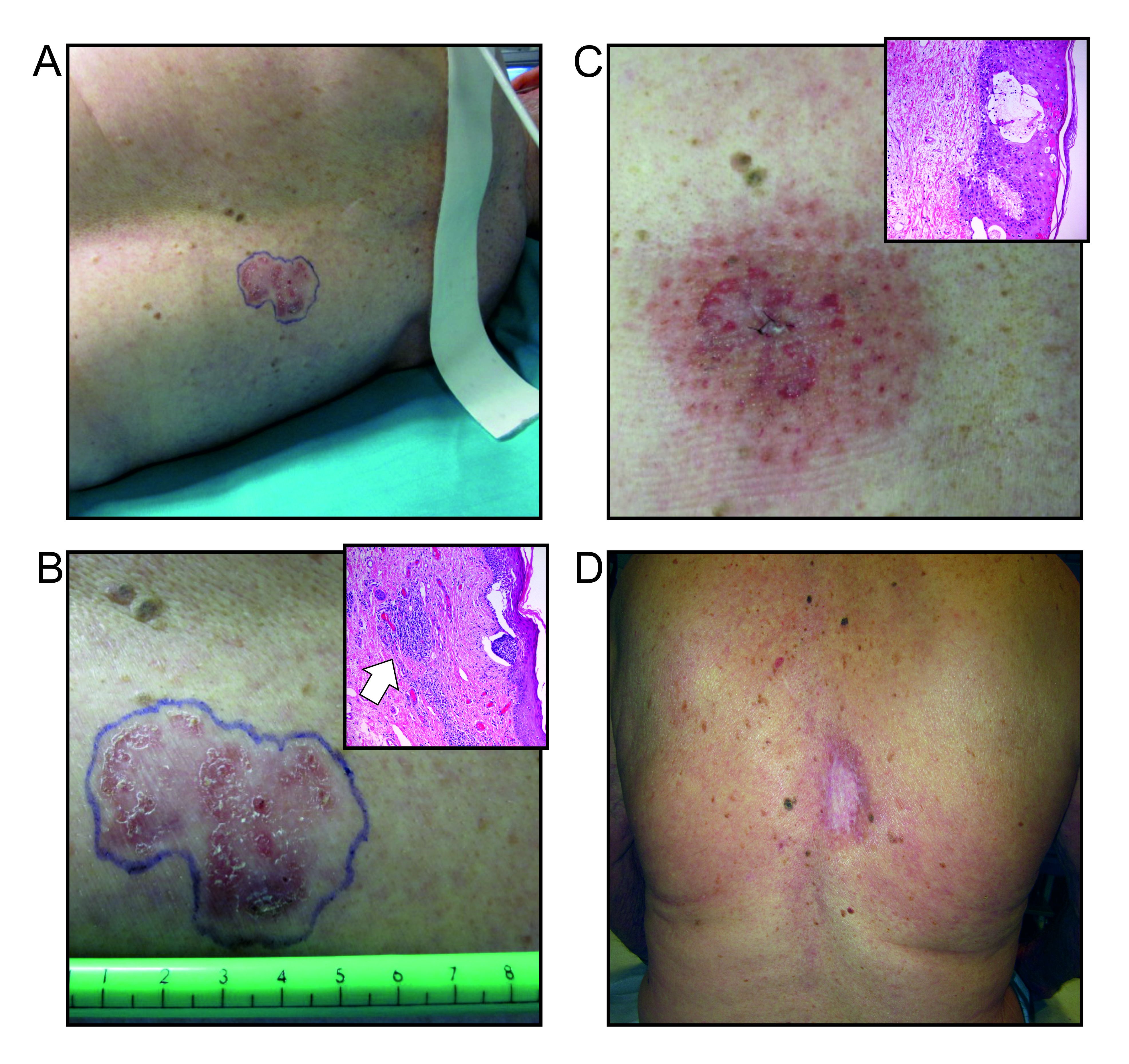

Supplement: Supplementary file 4 — Additional file 4: Figure S4. A primary basal cell carcinoma of the trunk in a patient with concomitant in-transit metastases from melanoma on the lower limb. Both basal cell carcinoma and lower limb in-transit metastases were simultaneously treated with electrochemotherapy; bleomycin was administered intravenously – according to the European Standard Operative Procedure of Electrochemotherapy (ESOPE)-, due to the concomitant presence of multiple in-transit metastases from melanoma. Baseline presentation (a, b); one month after electrochemotherapy (c); two-year and six-month follow-up showing long-lasting complete response (d). [file 12967_2017_1225_MOESM4_ESM.jpg]

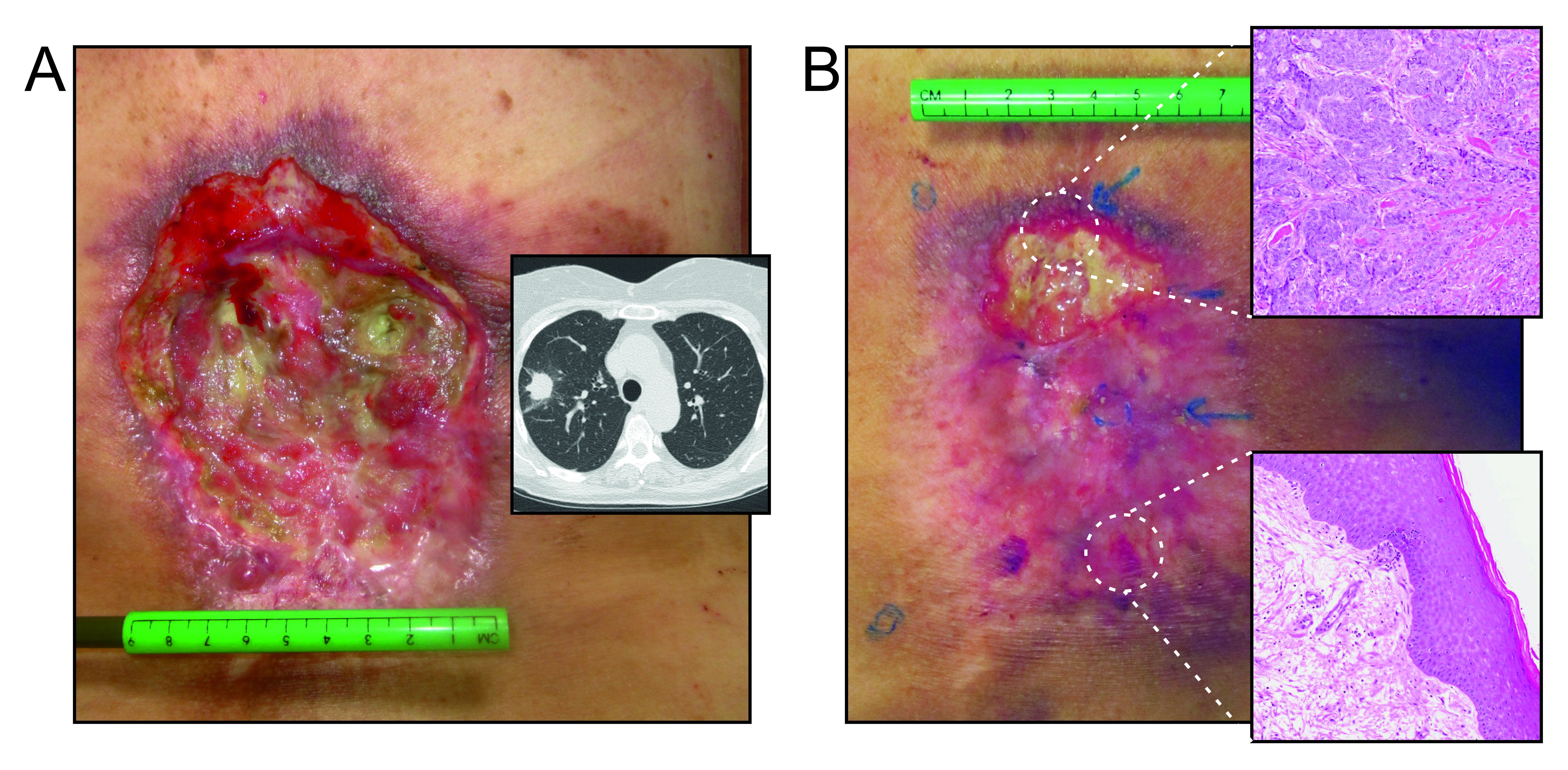

Supplement: Supplementary file 5 — Additional file 5: Figure S5. Recurrent, locally advanced basal cell carcinoma of the back in a 55 years-old woman with a synchronous lung metastasis. The latter was biopsied under CT-scan guidance. The pathology report indicated a metastasis from basosquamous carcinoma. The patient received anti-hedgehog therapy (vismodegib) with partial response; side effects associated with systemic treatment were tolerated for eight months and the patient finally refused further administrations. Therefore, stereotactic radiosurgery was performed on the lung metastasis. As to the basal cell carcinoma on the trunk, several surgical resections were previously attempted, also followed by external radiotherapy. The tumor was managed with four bleomycin-based ECT cycles, by using both intravenous and intratumoral chemotherapy. (a) Baseline presentation; (b) two-year follow-up indicating partial response with appreciable wound healing; tumor response was pathologically assessed by means of six punch biopsies, of which four confirmed tumor clearance (lower insert) and two showed residual disease (upper insert). [file 12967_2017_1225_MOESM5_ESM.jpg]
